# Design of Metasurface with Nanoslits on Elliptical Curves for Generation of Dual-Channel Vector Beams

**DOI:** 10.3390/nano11113024

**Published:** 2021-11-11

**Authors:** Xiaorong Ren, Manna Gu, Xiangyu Zeng, Rui Sun, Yuqin Zhang, Zijun Zhan, Lianmeng Li, Dawei Li, Hong Ma, Chuanfu Cheng, Chunxiang Liu

**Affiliations:** 1 College of Physics and Electronics, Shandong Normal University, Jinan 250014, China; bidud@126.com (X.R.); gumanna1996@outlook.com (M.G.); zengxiangyu0611@163.com (X.Z.); sunrui199812@163.com (R.S.); lilianmeng1234@163.com (L.L.); mahong@sdnu.edu.cn (H.M.); 2 Department of Physics, School of Electronic and Information Engineering, Qilu University of Technology (Shandong Academy of Sciences), Jinan 250353, China; 3 School of Science, Shandong Jianzhu University, Jinan 250101, China; ss_yghg@163.com; 4 Shanghai Institute of Optics and Fine Mechanics (SIOM), Chinese Academy of Sciences (CAS), Shanghai 201800, China; zhanzijun1990@live.cn (Z.Z.); lidawei@siom.ac.cn (D.L.)

**Keywords:** dual-channel focused vector beams, metasurfaces, geometric phase, propagation phase, ellipse

## Abstract

The manipulations of nanoscale multi-channel vector beams (VBs) by metasurfaces hold potential applications in various important fields. In this paper, the metasurface with two sets of nanoslits arranged on elliptic curves was proposed to generate the dual-channel focused vector beams (FVBs). Each set of nanoslits was composed of the in-phase and the out-of-phase groups of nanoslits to introduce the constructive interference and destructive interference of the output light field of the nanoslits, focusing the converted spin component and eliminating the incident spin component at the focal point. The two sets of nanoslits for the channels at the two focal points were interleaved on the same ellipses, and by setting their parameters independently, the FVBs in the two channels are generated under illumination of linearly polarized light, while their orders and polarization states of FVBs were controlled independently. The generation of the FVBs with the designed metasurfaces was demonstrated by the finite-difference time domain (FDTD) simulations and by the experimental verifications. The work in this paper is of great significance for the generation of miniaturized multi-channel VBs and for broadening the applications of metasurfaces.

## 1. Introduction

Vector beams (VBs) are light field distributions with spatially inhomogeneous polarizations [1], and they can be produced by superposing the orthogonal circularly polarized vortex states that couple the spin angular momentum (SAM) with the orbital angular momentum (OAM) of light [2]. Due to the unusual properties, VBs have provided many important applications in areas of both classical physics and quantum sciences. The classical applications include particle trapping [3], high resolution microscopy [4], optical encryption [5], particle acceleration [6], etc. Given the name of classical entangled light alternatively, VBs are the inseparable coupling of polarization and spatial modes, a property similar to the entanglement of quantum states [7], and they have been found quantum applications such as quantum information process [8] and quantum communications [9]. In particular, VBs have been developed as a novel resource of quantum information protocols to encode rotationally invariant qubits for alignment-free quantum communications over long distance [10]. To generate the VBs, researchers have proposed various methods over the last decade by using different traditional optical systems such as Michelson [11], Mach-Zehnder [12], Sagnac interferometers [13], and elements such as spatial light modulators [14], wave plates [15], Daman gratings [16], q-plates [17]. The generated versatile VBs include first-order and high-order VBs [18,19], vector beam arrays [20], high-order Poincaré beams [2], etc. However, these bulky optical systems often contain numerous optical elements and need complicated operations, which hinders the applications of VBs for miniaturized integrations.

Metasurfaces are inhomogeneous and anisotropic planar metamaterials composed of artificial sub-wavelength structures. With the flexible design of the nanostructure units, metasurfaces can arbitrarily modulate the phase, polarization, and amplitude of light, and have the unprecedented ability to manipulate light fields [20,21,22,23]. Metasurfaces have provided platforms for designing many novel compact devices, such as polarization conversion elements [22], broadband achromatic metalenses [23], and multiphoton quantum sources [24], which have facilitated the investigations for a series of fascinating physical phenomena [25]. Specifically, metasurfaces have been an effective tool to generate nanoscale VBs. Initially, in generations of VBs, the nanostructures in the metasurfaces are designed to directly control the polarization direction and phase of the output light fields [22,23,24,25,26], which results in practical inconveniences and limitations for metasurface designs. In 2016, F. Y. Yue et al. [27] proposed the metasurface to generate VBs by directly manipulating the superposition of two orthogonal circularly polarized vortex beams with different topological charges [2]. In principle, this method made use of the spin-orbit interactions in the metasurface nanostructures [28], and it has greatly simplified the implementation and has currently become the most popular method of metasurface design for the generating VBs [24,29,30,31,32,33,34]. During the last years, the generations of both multi-channel VBs [29,30,31,32] and FVBs [33,34] have been the subjects of particular interest, and they have been mainly realized by manipulating the superposition of two vortices of circular polarizations (CPs) through metasurface design. Furthermore, the focused multi- or dual-channel VBs are more useful, the generations are more challenging, and conspicuous progress has been achieved recently [35,36,37,38]. However, due to the conjugation of the geometric phase to left circular polarization (LCP) and right circular polarization (RCP), the metasurface using only the geometric phase for both the multi-channel and the focusing performances could lead to the unindependent generation of beams and result in the possible noise background by the conjugate divergent hyperbolic phase term [35]. For metasurfaces using the spatial multiplexing of area divisions for dual-channel operations and the resonant phase for focusing [36,37], the resultant asymmetric areas of the metasurface for each channel could cause the possible deteriorations of the VBs.

In this paper, we propose the metasurfaces with nanoslits arranged on ellipses for generation of independently controlled dual-channel FVBs. The basic principle is based on the sum of the optical paths from a nanoslit on an ellipse to the two foci is constant. The nanoslits of two groups on each ellipse are designed with the sums of the optical paths to be integer multiples of wavelength and odd integer multiples of half-wavelength, respectively. The nanoslits of two groups are alternately distributed with perpendicular orientations on the ellipses, and correspondingly, by the constructive interference, the converted spin component (CSC) in the output field is focused at the foci, and by the destructive interference, the incident spin component (ISC) is eliminated at the foci. Since the practical far-field observation plane is at a certain distance from the metasurface, the optical paths from the slits on an ellipse to longitudinally shifted focal points are changed, and we use two sets of the nanoslit groups that are interleaved on the same ellipses to compensate the path change. Simultaneously, the geometric phases of vortex are imposed on the two sets of nanoslits with respect to their corresponding focal points in the observation plane, and under illumination of the linearly polarization (LP), superposition of the focused vortex beams of orthogonal CPs is achieved at the focal point. Thus, the dual-channel FVBs are generated, and they are independently controlled with the spatial multiplexing of the nanoslits on the same elliptical curves. Essentially, the multiplexing made use of the propagation phase related to optical path, and it avoided the unindependent control of the VBs in different channels and the divergent hyperbolic phase factor due to the multiplexing with pure geometric phase [35], and also avoided deteriorations of the VBs due to the spatial multiplexing of area divisions [36,37]. Theoretically, we gave the mathematical analysis for the design principle and the light fields of the VBs around the two focal points in the observation plane. With the finite-difference time domain (FDTD) method, we performed the simulations and optimizations of metasurfaces to generate respectively, the two identical FVBs, standard and π-phase [39] FVBs of the same order, FVBs of different orders in dual-channels. Experimentally, we fabricated the metasurface samples and generated the different dual-channel VBs of satisfactory qualities, which demonstrated the feasibility of the proposed metasurfaces. As the foundation for generating multi-channel VBs and arrayed VBs, our method of dual FVB generation would be of significance for manipulations of more generalized VBs. Furthermore, it would be important for achieving compact and miniaturized VB generator devices, and would hold potential applications in areas such as information encoding and transmission [40], OAM multiplexing communications [41], quantum information processing [8].

## 2. Principles of the Metasurface Design and Vector Beam Generations

**Figure 1:**
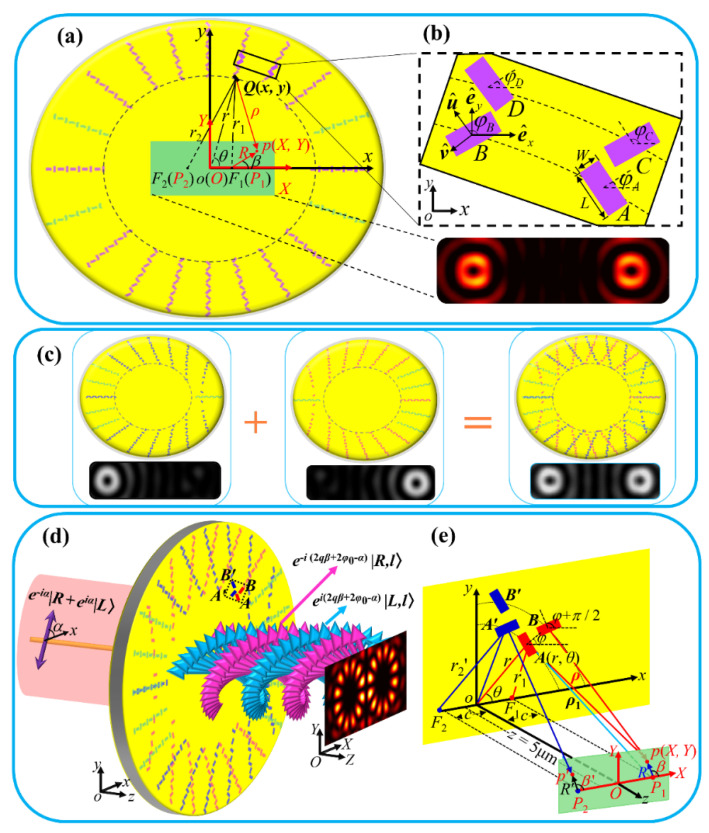
Schematic geometry for plasmonic metasurface based on the elliptic curve to generate dual-channel FVBs. (**a**) Schematic of a metasurface composed of the nanoslits to generate the in-plane dual-channel FVBs at the two foci. (**b**) Enlarged view and geometry of the nanoslits at the in-phase point and the out-of-phase point on two adjacent ellipses. (**c**) Design principle for metasurface generating dual-channel FVBs in observation plane at a certain distance away from the metasurface. (**d**) Schematic for generating dual-channel FVBs under illumination of linearly polarized light. (**e**) Geometry for light propagations in dual-channel FVBs generation.

Figure 1 shows the schematic geometry and the principle for generating the dual-channel FVBs by the plasmonic metasurface based on the elliptical curves. We start our analysis from geometry to generate FVBs with cores at the elliptical foci in central area in the metasurface plane, as shown in Figure 1a. Later, we will extend the analysis to the case of the observation plane at the distance *z* from the metasurface, as shown in Figure 1b–e.

In Figure 1a, a gold film of thickness 200 nm is coated on the SiO_2_ substrate, the rectangular nanoslits with length *L* and width *W* are drilled in the film. The nanoslit at point *Q* (*x*, *y*) in the object coordinate system *oxy* is also specified by *Q* (*r*, *θ*), with *x* = *r*cos *θ*, and *y* = *r*sin *θ*. The nanoslits are arranged on 2*m* ellipses, all the ellipses share the common foci *F*_1_ and *F*_2_, and the distance between the foci is 2*c*. The semi-major and semi-minor axes of the *j*-th ellipse are a*_j_* and *b_j_*, respectively, with *b_j_* = (*a_j_*^2^ − *c*^2^)^1/2^. The generated FVBs at *F*_1_ and *F*_2_ are observed in the central area of the metasurface, and an observation point *p* is specified by *p* (*X*, *Y*) in observation coordinate system *OXY*, or by *p* (*R*, *β*) with *X* = *c* + *R*cos *β*, and *Y* = *R*sin *β*, where *c* is the distance from the ellipse center to a focus. We notice that the origins *O* and *o* of the observation and object coordinate systems coincide. The position of a nanoslit on an ellipse is so determined that its distances *r*_1_ and *r*_2_ to the two focal points *F*_1_ and *F*_2_ satisfy *r_j_*_1_ + *r_j_*_2_ = 2*a_j_* = [(*N*_0_ − 1) + *j*]*λ*, where λ is the wavelength of the illuminating light, *N*_0_ is an even number, and *j* = 1, 2, 3, etc. This indicates that the major axis 2*a*_1_ of the first ellipse is *N*_0_*λ*, and we set *r_j_*_1_ and *r_j_*_2_ for each nanoslit to both integer multiples of *λ*/2. The orientation angle *φ* of the nanoslit is defined as the angle of the direction perpendicular to the slit with respect to *x*-axis, and it is set *φ* = *qθ* + *φ*_0*j*_ and *φ* = *qθ* + *φ*_0*j*_ + *π*/2 for the nanoslit, respectively, according to its *r*_1_ being even and odd integer multiples of *λ*/2, where *q* is the rotation order of the nanoslit, *θ* is the azimuth angle, and *φ*_0j_ is the initial orientation angle for the slit at *θ* = 0 on the *j*-th ellipse. Besides, on the *j*-th and (*j* + 1)-th ellipses, *φ*_0*j*+1_ − *φ*_0*j*_ = π/2, so that a pair of nanoslits at corresponding positions with the same *θ* on the adjacent ellipses are perpendicular to each other. It is well understood that the long and thin rectangular nanoslit composing of the metasurface can be regarded as a polarizer [42,43]; this is resulted from the polarization response of the nanoslit to incident light, while the intensity transmittance |*T_u_*|^2^ of a nanoslit for the wave linearly polarized in the direction perpendicular to the longer side of the nanoslit is much greater than the transmittance |*T_v_*|^2^ for that parallel to the longer side of the nanoslit, i.e., |*T_u_*|^2^ ≫ |*T_v_*|^2^. Here, the unit vectors perpendicular and parallel to the longer side of the nanoslit are defined as $\hat{u}=\cos\varphi {\hat{e}}_{x}+\sin\varphi {\hat{e}}_{y}$ and $\hat{v}=-\sin\varphi {\hat{e}}_{x}+\cos\varphi {\hat{e}}_{y}$, respectively, with ${\hat{e}}_{x}$ and ${\hat{e}}_{y}$ the unit vectors in *x*- and *y*- directions, respectively, as shown in Figure 1b. The Jones matrix for the nanoslit as a polarizer with the transmitted axis at angle *φ* is given by

(1) $$J(\varphi )=\left[\begin{array}{cc} \cos^{2}\varphi & \sin\varphi \cos\varphi \\ \sin\varphi \cos\varphi & \sin^{2}\varphi \end{array}\right]$$

When the metasurface is illuminated by the circularly polarized light $E_{in}^{\sigma }=({\hat{e}}_{x}+i\sigma {\hat{e}}_{y})/\sqrt{2}$, where helicities *σ* = 1 and *σ* = −1 represent light of RCP and LCP, respectively, the transmitted light field is written as:

(2) $$E_{out}=J(\varphi )E_{in}^{\sigma }=E_{out}^{\sigma }+E_{out}^{-\sigma }=\frac{1}{2\sqrt{2}}({\hat{e}}_{x}+i\sigma {\hat{e}}_{y})+\frac{1}{2\sqrt{2}}({\hat{e}}_{x}-i\sigma {\hat{e}}_{y})e^{i2\sigma \varphi }$$

where $E_{out}^{\sigma }$ and $E_{out}^{-\sigma }$ are the ISC and the CSC in the transmitted light, with helicities *σ* and −*σ*, respectively, and 2*σφ* is the geometric phase.

Now we first consider the light field *E* (*X*, *Y*) at observation point *p* (*X*, *Y*) in the metasurface plane at *z* = 0. On the *j*-th ellipse, the initial nanoslit is at point (*x* = *a_j_*, *y* = 0), where *a_j_* is the semi-major axis of the ellipse, while its distances to the focal points *F*_1_ and *F*_2_ are *r*_10_ = *a_j_* − *c* and *r*_20_ = *a_j_* + *c*, respectively. For nanoslit at a point with distance *r*_1_ to focal point *F*_1_ being *r*_1_ = *r*_10_ + *nλ*, with integer number *n* > 0, the propagation phase corresponding to *r*_1_ is the same as that of the initial slit, this causes the constructive interference of the light field at *F*_1_, and such point (slit) in the ellipse is called the in-phase point (slit) with respect to focal point *F*_1_. For the nanoslit at a point with the distance to focal point *F*_1_ being *r*_1_ = *r*_10_ + (2*n*−1)*λ*/2, its light field is of destructive interference with that of the initial slit, and the point (slit) is called out-of-phase point (slit) with respect to *F*_1_. Thus, in each ellipse, the two groups of nanoslits are alternately arranged at the in-phase and the out-of-phase points with respect to *F*_1_, and the initial constants of orientation *φ*_0*j*_ and *φ*_0*j*_ + π/2 are set for orientation angle *φ* of nanoslits in the in-phase and out-of-phase groups, respectively, so that two adjacent slits can be regarded as being perpendicular to each other. For nanoslit in the in-phase group, the distances *r*_10_ + *nλ* and *r*_20_ − *nλ* to foci *F*_1_ and *F*_2_ are both integer multiples of the wavelength, while for slit in the out-of-phase group, the distances *r*_10_ + (2*n* − 1)λ/2 and *r*_20_ − (2*n* − 1)*λ*/2 to foci *F*_1_ and *F*_2_, are both odd multiples of half the wavelength. We have:

(3) $$\begin{array}{l} (r_{1}+n\lambda )+(r_{2}-n\lambda )=2a_{j}\\ {}[r_{1}+(2n-1)\lambda /2]+[r_{2}-(2n-1)\lambda /2]=2a_{j} \end{array}$$

Then, the propagation phases of the output fields from a pair of adjacent nanoslits *A* and *B* as in Figure 1b result in destructive interference of the light fields at both foci *F*_1_ and *F*_2_, respectively. Considering that the two nanoslits take the orientations perpendicular to each other, the relative geometrical phase factor *e^iσπ^* in their light fields is introduced. Based on Equation (2), the ISC $E_{out}^{\sigma }$ for slit *A* and slit *B* are eliminated due to the destructive interference at foci *F*_1_ and *F*_2_, while the CSC $E_{out}^{-\sigma }$ for the two nanoslits are of constructive interference at the foci due to the combination of the opposite propagation phase and opposite geometrical phase. Resultantly, the superimposed light field of the two slits are derived as:

(4) $$E_{out}^{j,j}=\frac{1}{2\sqrt{2}}({\hat{e}}_{x}-i\sigma {\hat{e}}_{y})e^{i2\sigma \varphi_{0j}}(e^{i2\sigma q\theta_{A}}+e^{i2\sigma q\theta_{B}})$$

where *θ_A_* and *θ_B_* are the azimuth angles of slit *A* and slit *B*, respectively, and the superscript *j*, *j* denotes the superposition of two slits in the *j*-th ellipse. While on the (*j* + 1)-th ellipse, nanoslit *C* at the same azimuth angle *θ* as nanoslit *A* also in the different slit groups, and nanoslits *C* and *A* also form a pair of orthogonal slits. Considering the propagation phase and geometric phase, we have superposed light field of the two slits on the (*j* + 1)-th and *j*-th ellipses:

(5) $$E_{out}^{j,j+1}=\frac{1}{2\sqrt{2}}({\hat{e}}_{x}-i\sigma {\hat{e}}_{y})e^{i2\sigma (q\theta_{A}+\varphi_{0j})}$$

where *θ_A_* = *θ_C_* is used. Equations (4) and (5) manifest that the ISC in the transmitted light field is eliminated with the destructive interference, while the CSC is maximized by the constructive interference with combination of the geometric phase and the propagation phase. Equation (5) will be used in the following derivations of the light field produced by the metasurface.

With the right focus *F*_1_ as the example, we first analyze the light field near the focus in the metasurface plane. As shown in Figure 1a, and based on the Huygens-Fresnel principle [44], the superimposed light field produced by all the nanoslits in the *j*-th and the (*j* + 1)-th ellipses at the observation point *p* (*X*, *Y*) near *F*_1_ is:

(6) $$E_{F1}^{j,j+1}(X,Y)=-(i/\sqrt{\lambda })\int_{l}dlE_{out}^{j,j+1}e^{i(k\rho_{j}+\pi /4)}/\sqrt{\rho_{j}}$$

where *k* = 2*π/λ*, *ρ_j_* = [*r_j_*_1_^2^ + *R*^2^ − 2*Rr_j_*_1_cos (*θ* − *β*)]^1/2^ is distance from *Q* to point *p.* In the observation area near the center *O*, we have *R* ≪ *r*_1_. In the exponential term of the above equation, *ρ_j_* ≈ *r_j_*_1_ − *R* cos(*θ* − *β*), while the factor $1/\sqrt{\rho_{j}\;}\approx 1/\sqrt{r_{j1}}$, then above equation is written as:

(7) $$E_{F1}^{j,j+1}(X,Y)=-(ie^{i\pi /4}/\sqrt{\lambda })\;\int_{l}dlE_{out}^{j,j+1}e^{ik[r_{j1}-R\cos(\theta -\beta )]}/\sqrt{r_{j1}}$$

Further, the light field produced by the metasurface is written as:

(8) $$U_{F1}(X,Y)=-(ie^{i\pi /4}/2\sqrt{2\lambda })\;\sum_{j\in G}^{}\int_{l}dl({\hat{e}}_{x}-i\sigma {\hat{e}}_{y})e^{i2\sigma \varphi_{j}}e^{ik[r_{j1}-R\cos(\theta -\beta )]}/\sqrt{r_{j1}}$$

where *G* = [1, 3, …, 2*m* − 1]. The analytical integral along an ellipse path *l* in the above equation is unsolvable. For a simple understanding, we can make an analogy of the case to that of nanoslits arranged in a circular ring [20], in which, the integral along the circular path is the Bessel vortex beam of *e^i^*^2*σqβ*^*J*_2*σq*_ (*k R*) with the doughnut profile as Bessel function *J*_2*σq*_·(*k*·*R*) of order 2*σq*; while for metasurfaces with nanoslits on multiple circular rings, the doughnut profile *ψ*′(*R*) is related to the confluent hyper geometrical function _1_*F*_2_[*a_h_*, *b_h_*, *c_h_*; *x*], i.e., *ψ*′(*R*)∝ *R*^2*q*^*·*_1_*F*_2_[*q* + 1, *q* + 2, 2*q* + 1; −(*kR*)^2^] [45,46], and the corresponding vortex beam is *ψ*′(*R*)*e^i^*^2*σqβ*^. Although it is obvious that the integral along the ellipses in the above equation may have the different doughnut from the confluent hyper geometrical function *ψ*′(*R*), ***U****_F_*_1_(*X*, *Y*) is represented as a vortex beam *ψ*(*R*)*e^i^*^2*σqβ*^, where *ψ*(*R*) expresses the doughnut profile for the multiple ellipses, and *e^i^*^(2*σqβ+φ*^^0*j*)^ represents vortex phase-front from the factor *e^i^*^(2*σqθ+φ*^^0*j*)^ inside the integral. Then, Equation (8) is written as:

(9) $$U_{F1}(X,Y)=A\;\left[\begin{array}{c} 1\\ -\sigma i \end{array}\right]e^{i2\sigma (q\beta +\varphi_{0j})}\psi (R)$$

where *A* is a complex constant, and *e^i^*^(2*σqβ+φ*^^0*j*)^ is vortex phase around focus *F*_1_. The above equation indicates that the vortex beam of CP with helicity −*σ* is formed.

Similarly, the above analysis is also applicable to the left focal point *F*_2_. Under illumination of circularly polarized light, it is possible to simultaneously generate the vortex beams ***U****_F_*_1_(*X*, *Y*) and ***U****_F_*_2_(*X*, *Y*) near foci *F*_1_ and *F*_2_, respectively, in the metasurface plane at *z* = 0, and this indicates that the generation of dual-channel vortices can be realized. In the case when light of LP is used to illuminate the metasurface, it is equivalent to the simultaneous illumination of LCP and RCP, and the two converted RCP and LCP components are superimposed at the area near each focus, with FVBs formed at the two foci.

Based on the principles for generating the in-plane dual-channel vortices, we extend the design of the metasurface to the case when the observation plane is away from the metasurface. As shown in Figure 1e, in the observation plane *OXY* with longitudinal coordinate *z*, the generated dual vortex beams are expected to focus at the same focal points *P*_1_(*c*, 0, *z*) and *P*_2_(−*c*, 0, *z*) in the *OXY* plane, respectively. The distance from a slit in an ellipse to a focal point is *ρ_i_* = (*r_i_*^2^ + *z*^2^)^1/2^, where *i* = 1, 2, and *r_i_* = *a_j_* ± *c* cos θ. However, due to the addition of *z*, the propagation phases of the nanoslits at the in-phase and the out-of-phase points cannot satisfy simultaneously the conditions of constructive and destructive interferences at the two focal points *P*_1_ and *P*_2_. To this end, we design two sets of nanoslits: One set of the slits, as those drawn in red in Figure 1c, are called right-channel nanoslits, of which the initial slit point (*a_j_*, 0) is either the in-phase or out-of-phase point with respect to the focal point *P*_1_; the other set of slits, as drawn in blue, are called the left channel nanoslits, with their initial slit point (−*a_j_*, 0) is the in-phase or out-of-phase with respect to the focal point *P*_2_. The parameters (*q_l_*, *φ_l_*_0_) and (*q_r_*, *φ_r_*_0_) for the nanoslits in the left and right channels can be set independently, where *q_l_*, *q_r_* are the rotation orders, and *φ_l_*_0_, *φ_r_*_0_ are the initial orientation angle, respectively. Correspondingly, the independently controlled vortex beams are generated at the two focal points. The light field produced by the right channel nanoslits is focused at the right focus *P*_1_, and it is denoted as ***U****_P_*_1_(*X*, *Y*); although to some degree ***U****_P_*_1_(*X*, *Y*) may have influence on the light field at the left focus *P*_2_, it is insignificant in comparison with the focused light field ***U****_P_*_2_(*X*, *Y*) produced by the left channel nanoslits at *P*_2_. Thus, the crosstalk between the fields of the two channels may be neglected, as is demonstrated in the results in the later sections. For the nanoslits to be arranged on the ellipses more homogeneously, and for better vector beams to be generated, we added complementary nanoslits of equal number to in-phase and out-of-phase groups in the two sets of nanoslits, as those shown in bean-green in Figure 1. It is noted that the sum of the optical paths from a complementary nanoslit to the two focal points is still integer multiples of wavelength or odd integer multiples of half-wavelength, respectively, and the elliptical curve on which the nanoslits were arranged had the semi-major axis a*_j_*′, with *a_j_* < *a_j_*′ < *a_j_*_+1_ and *a_j_*′ = [*ρ*^2^ − *z*^2^]^1/2^ ∓*c*cos *θ*. Here, *θ* was set at the value for complementary nanoslits to be at middle of the original in-phase and out-of-phase points with larger gaps.

With the conditions of the constructive and destructive interferences, the semi-major axis *a_j_* is changed as *a_j_* = {[(*a*_1_ ± *c*)^2^ + *z*^2^]^1/2^ + (*j* − 1) *λ*/2 − *z*^2^}^1/2^ ∓*c*, and accordingly, the elliptical curves, the positions of initial slits, and the in-phase and out-of-phase points for each set of slits are also modified. For the set of slits in red corresponding to the right focus *P*_1_, *ρ_j_* in Equation (6) is written as *ρ_j_* = [*r_j_*_1_^2^ + *z*^2^ + *R*^2^ − 2*R* (*r_j_*_1_^2^ + *z*^2^)^1/2^ cos(*θ* − *β*)]^1/2^, and the light field ***U****_P_*_1_(*x*, *y*) near the focal point *P*_1_ similar to Equation (9) is changed as

(10) $$U_{P1}(X,Y)=C\left[\begin{array}{c} 1\\ -\sigma i \end{array}\right]e^{i2\sigma (q_{r}\beta +\varphi_{r0})}\;\psi_{1}(R)$$

where *C* is complex constant, and subscript *r* represents parameters of the nanoslits of the right channel. Similarly, the light field of the vortex beam produced by the other set of slits (in blue) with core at the left focus *P*_2_ is rewritten as:

(11) $$U_{P2}(X,Y)=C^{\prime }\left[\begin{array}{c} 1\\ -\sigma i \end{array}\right]e^{i2\sigma (q_{l}\beta \prime +\varphi_{l0})}\;\psi_{2}(R^{\prime })$$

where *C*′ the complex constant conjugate to *C*, while subscript *l* represents parameters of the left channel. By adding the light fields ***U****_P_*_1_(*X*, *Y*) and ***U****_P_*_2_(*X*, *Y*) given in Equations (10) and (11), we have the light field of the dual-channel vortex beams:

(12) $$U(X,Y)=U_{P1}(X,Y)+U_{P2}(X,Y)$$

Equations (10)–(12) indicate that under the incidence of circularly polarized light, the vortex beams with order 2*q_l_* and 2*q_r_* are generated at two focal points *P*_2_ and *P*_1_ in the observation plane, respectively, and the chirality of vortex beams is opposite to that of the incident light.

Now, we analyze the principle for generating dual FVBs under illumination of linearly polarized light $E_{in}^{L}$, which contains the LCP and RCP:

(13) $$E_{in}^{L}=E_{0}\left[\begin{array}{c} \cos\alpha \\ \sin\alpha \end{array}\right]=\frac{E_{0}}{2}\left[\begin{array}{c} 1\\ i \end{array}\right]e^{-i\alpha }+\frac{E_{0}}{2}\left[\begin{array}{c} 1\\ -i \end{array}\right]e^{i\alpha }$$

where *α* is the polarization angle of the incident light with respect to *x*-axis, *E*_0_ is the amplitude of incident light. Using Equations (10)–(13), and remembering *σ*, takes both values of 1 and −1, we have the light field of the dual-channel FVBs in the observation plane:

(14) $$\begin{array}{ll} U_{vc}(X,Y) & =C\psi_{1}(R)\left\{\left[\begin{array}{c} 1\\ i \end{array}\right]e^{-i(2q_{r}\beta +2\varphi_{r0}-\alpha )}+\left[\begin{array}{c} 1\\ -i \end{array}\right]e^{i(2q_{r}\beta +2\varphi_{r0}-\alpha )}\right\}\;\\ & +C^{\prime }\psi_{2}(R^{\prime })\left\{\left[\begin{array}{c} 1\\ i \end{array}\right]e^{-i(2q_{l}\beta^{\prime }+2\varphi_{l0}-\alpha )}+\left[\begin{array}{c} 1\\ -i \end{array}\right]e^{i(2q_{l}\beta^{\prime }+2\varphi_{l0}-\alpha )}\right\} \end{array}$$

where the subscript *vc* of ***U****_vc_* represents vector beam. The term in the first brace in the right side of above equation represents a linear polarized VB of order 2*q_r_* in the right channel at focal point *P*_1_, and it is the equally-weighted superposition two conjugate eigen states of total angular momentum, each of which is a vortex state *e^±i^*^2*σqrβ*^ carried by the light waves of LCP or RCP [2]. While the term in the second brace is the linear polarized VB of order 2*q_l_*. Equation (14) demonstrates that the polarization state of the VB in each channel determines the polarization distribution in the across transverse plane, and it depends on the initial orientation angle *φ_l_*_0_, *φ_r_*_0_, and the incident polarized angle *α* [2].

From the analysis of the metasurface design, we see that the two sets of nanoslits symmetrically are interleaved on the same ellipses, they are not spatially overlapped, and the crosstalk between the two channels are avoided. The parameters of two sets of nanoslits are independently set, and the FVBs in each channel can be flexibly controlled. Therefore, our method of dual-channel FVBs generation is different from those previously reported in the literature, such as the methods for VB generation in different channels with unindependent control, and with the spatial multiplexing of area divisions, which may induce inconvenience in controlling the VBs and deterioration of the VB quality.

## 3. Results of Simulations

Based on Equation (14), the linear polarization states of dual channel FVBs can be controlled either by initial angles *φ_l_*_0_ and *φ_r_*_0_ of metasurfaces, or by polarization angle *α* of incident light. When the incident light is horizontally polarized with *α =* 0°, the radially polarized FVBs are generated in dual-channels for metasurface with *φ_l_*_0_ = *φ_r_*_0_ = 0°, and two azimuthally polarized FVBs are generated for metasurface with *φ_l_*_0_ = *φ_r_*_0_ = 45°. While for a specific sample with constant *φ_l_*_0_ and *φ_r_*_0_, the polarization states of the FVBs can be adjusted by changing the polarization angle *α* of incident light. For example, when the vertical polarized light illuminates the above metasurface with *φ_l_*_0_ = *φ_r_*_0_ = 0°, the azimuthal polarized VBs can also be generated. It is interesting to note that while the absolute values of the rotation orders *q_l_* and *q_r_* determine the orders 2*σ|q_r_*| and 2*σ|q_l_|* of FVBs, the positive and negative signs of *q_l_* and *q_r_* control the rotation direction of the polarization distribution of the FVBs. Under the same conditions in which the radially and azimuthally polarized FVBs are generated for metasurface with *q_l_* > 0 and *q_r_* > 0, as discussed above, the π-radially and π-azimuthally polarized FVBs [2,39] are generated for metasurfaces with *q_l_* < 0 and *q_r_ <* 0.

We designed various metasurfaces for generating different dual-channel FVBs. The method of FDTD was used for the simulations of the light fields and the optimizations of the metasurfaces. Based on the results of parameter sweep for a gold rectangular nanoslit, which indicates that in the dimension range of nanoslit length *L* = 180~300 nm and width *W* = 70~120 nm, the intensity transmittance ratio |*T_u_*|^2^/|*T_v_*|^2^ is about or over a multiple of two orders for a nanoslit with a ratio of *L* to *W* over 2.5, we have a good room for choosing a nanoslit as a polarizer. We practically performed the simulations of the VBs generated by several metasurfaces composed of the applicable nanoslits, and considering the optimized quality of the generated VBs, the nanoslit with *L* = 300 nm and *W* = 100 nm are finally selected. The wavelength of the incident light is 632.8 nm, and the distance of the observation plane from the metasurface is 5 μm. A metasurface contains ten ellipses, and the focal length of all ellipses in a metasurface is set as *c* = 1.2 μm. The semi-major axis of the innermost ellipse is *a*_1_ = 5.2 μm. For each sample, we also conducted the numerical integration of the generated dual-channel FVBs based on theoretical results given by Equations (6), (7) and (14).

**Figure 2:**
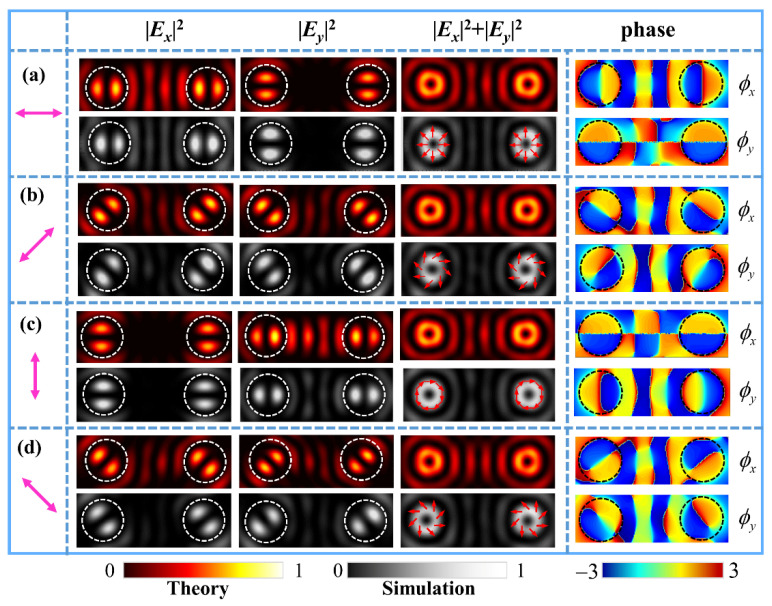
Theoretical and simulated results of the same FVBs of orders *l_l_* = *l_r_* = 1 and the corresponding simulated phase distributions of *x*- and *y*-components in the dual channels in the observation plane under the illumination of linearly polarized light with polarization angles at (**a**) *α =* 0°, (**b**) *α =* 45°, (**c**) *α =* 90°, and (**d**) *α =* 135°, respectively. The purple double arrows indicate the polarization direction of the incident light. The arrows on the simulated intensity patterns in the right column schematically show the polarization distributions of the VBs.

We first performed the simulations of a metasurface for generating two identical first-order FVBs of fundamental polarization states. The parameters of the metasurface were set correspondingly as (*q_l_*, *φ_l_*_0_; *q_r_*, *φ_r_*_0_) = (0.5, 0°; 0.5, 0°). Figure 2a–d shows the theoretical results of the numerical integration and FDTD simulation results of the different FVBs in the dual channels. The figures from top to bottom are the intensity patterns of the FVBs of radial, 45°-slanted, azimuthal, and 135°-slanted polarizations, respectively, generated by the metasurface under the illuminating light polarizing in horizontal, 45°, vertical, and 135° directions, as marked by the magenta double arrows. The patterns from left to right are, respectively, the component intensities of |***E****_x_*|^2^, |***E****_y_*|^2^, the total intensities of |***E****_x_*|^2^ + |***E****_y_*|^2^ and the phase maps of *x*- and *y*-components of the corresponding VBs. The petal-like patterns of |***E****_x_*|^2^ and |***E****_y_*|^2^ for the *x* and *y* components are the reflection of the polarization states of first-order VBs. These results demonstrate that the dual-channels FVBs are well generated with the designed metasurface.

**Figure 3:**
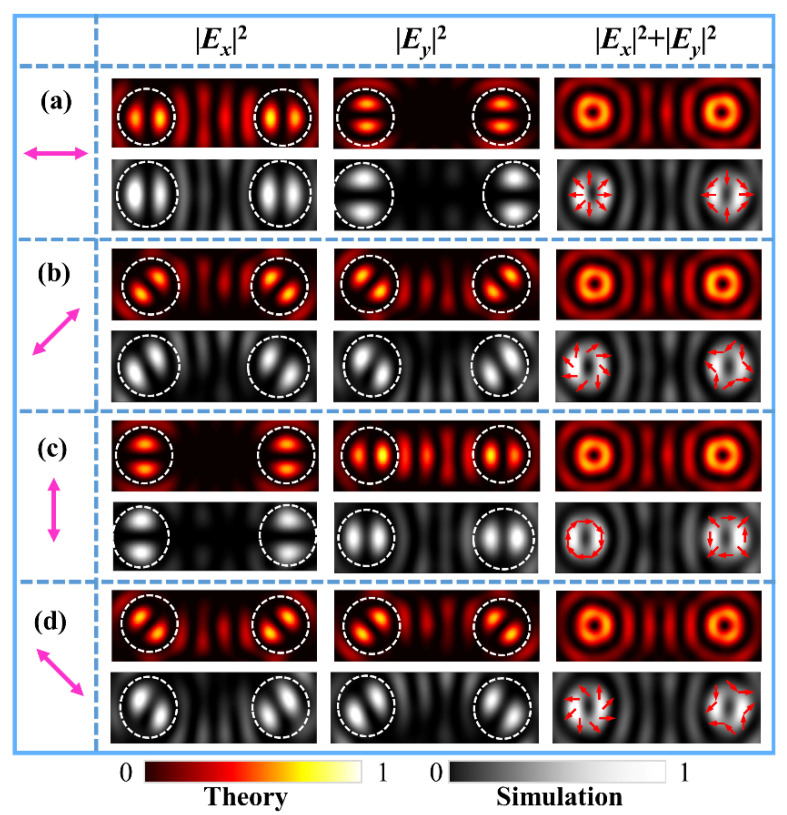
Theoretical and simulated intensity patterns of different FVBs of orders *l_l_* = 1 (VBs) and *l_r_* = −1 (π − VBs) in the dual channels under the illuminating light polarizing at 0° (**a**), 45° (**b**), 90° (**c**), and 135° (**d**), respectively. The magenta double arrows represent the polarization direction of illumination light.

Next, we generate the FVBs with different polarization states in the two channels. We designed a metasurface sample with the parameters (*q_l_*, *φ_l_*_0_; *q_r_, φ_r_*_0_) = (0.5, 0°; −0.5, 0°). With *q_l_ = −q_r_*, the first-order radial (azimuthal) and π-radial (azimuthal) FVBs are generated, respectively, at the left and right focal points under the illuminating light of horizontal (vertical) polarization. The theoretical results of numerical integrals and FDTD simulation results are shown in Figure 3a,c. The magenta double arrows represent the polarization direction of illuminating light. Figure 3b and Figure 3d are, respectively, results of generated 45°- and π-45° slanted, and 135°- and π-135°-slanted FVBs of the first order in the dual channels, respectively, produced by the metasurface under illumination of 45° and 135° polarizations. The schematics of the polarization states are drawn on the doughnut profiles of the total light intensity patterns. It is interesting to notice that in Figure 3a,c, the intensity petal patterns of the FVBs and π-FVBs are the same, their polarization states as drawn on the doughnut profiles of the total light intensities are different. It is more interesting that in Figure 3b,d, the intensity petals of FVBs and π-FVBs are in the perpendicular orientations, demonstrating obviously the difference in a VB and its corresponding π-VB.

**Figure 4:**
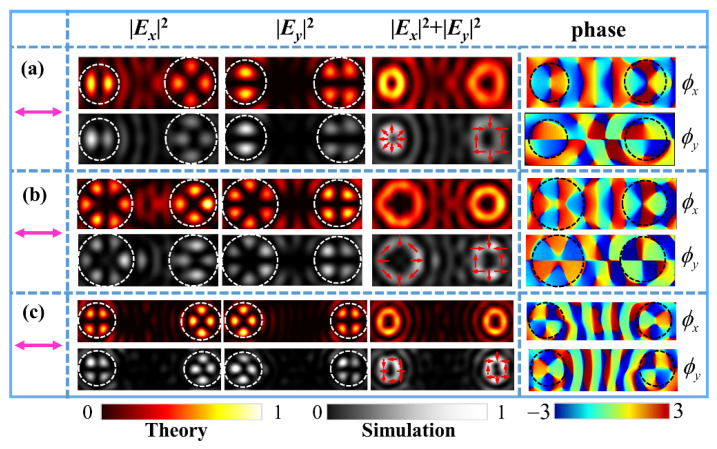
The intensity patterns of different FVBs of the theoretical results and FDTD simulations under horizontally polarized light illumination and the corresponding theoretical phase distributions of *x*- and *y*-components. (**a**) The radially polarized dual-channel FVBs of orders *l_l_* = 1 and *l_r_* = 2. (**b**) The radially polarized FVBs of orders *l_l_* = 3 and *l_r_* = 2. (**c**) The azimuthally polarized and radially FVBs of order 2 in the left and right channels.

We also designed three metasurface samples with |*q_l_*| ≠ |*q_r_*| to demonstrate the generation of FVBs of different orders or different polarization states in the two channels. The theoretical results and FDTD simulation results of intensity patterns for the metasurfaces under illumination of horizontal polarization are shown in Figure 4. The theoretical phase maps of *x*- and *y*-components are also shown, respectively, in the rightmost column of Figure 4. Specifically, Figure 4a shows the radially polarized FVBs of order *l_l_* = 1 in the left channel and order *l_r_* = 2 in the right channel produced by the metasurface sample with parameters (*q_l_*, *φ_l_*_0_; *q_r_, φ_r_*_0_) = (0.5, 0°; 1, 0°). Figure 4b shows the radial FVBs of order *l_l_* = 3 and order *l_r_* = 2 generated the metasurface sample with parameters (*q_l_*, *φ_l_*_0_; *q_r_, φ_r_*_0_) = (1.5, 0°; 1, 0°). It can be seen that higher order FVBs have bigger doughnut profiles, which can be analogous to that Bessel function of higher order has larger value of first root. Figure 4c shows the azimuthally polarized FVBs of order *l_l_* = 2 and radially polarized FVBs of order *l_r_* = 3 by the metasurface sample with the parameters (*q_l_*, *φ_l_*_0_; *q_r_, φ_r_*_0_) = (1, 45°; 1, 0°). Here, we note that initial angle *φ_l_*_0_ = 45° corresponds to the generation of the azimuthally polarized FVB under illumination of horizontal polarization. In order to avoid the crosstalk between the two channels and generate higher quality VBs, the focal length of the selected elliptic curves was increased to *c* = 2.2 μm when designing this metasurface.

**Figure 5:**
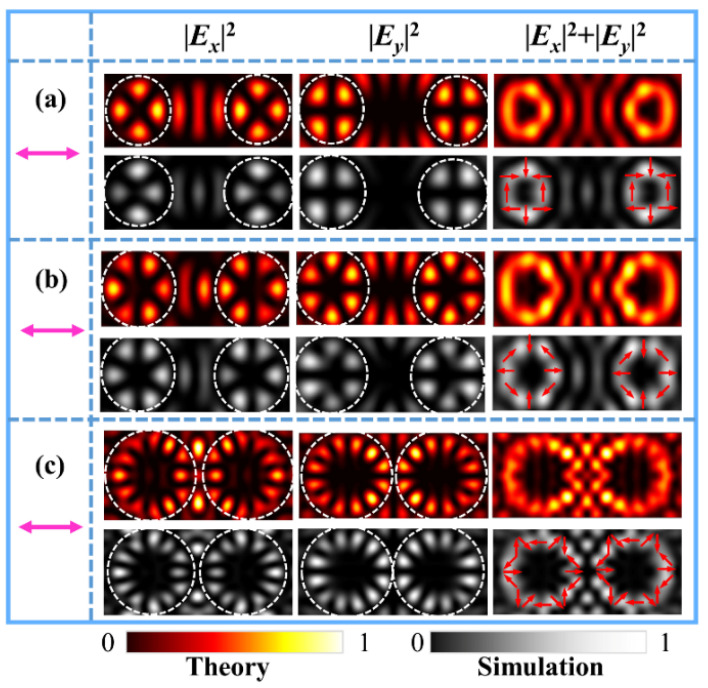
(**a**–**c**) The theoretical and FDTD simulated results of the *x*-, *y*-component, and the total intensities of the FVBs produced by metasurfaces with parameters (*q_l_*, *φ_l_*_0_; *q_r_, φ_r_*_0_) = (1, 0°; 1, 0°), (1.5, 0°; 1.5, 0°), and (3, 0°; 3, 0°), respectively, under illumination of horizontally polarized light. The purple double arrows indicate the polarization direction of the incident light, and the arrows on the intensity doughnuts represent the schematic polarization distributions.

For further demonstrations, we also designed the metasurfaces with the parameters (*q_l_*, *φ_l_*_0_; *q_r_, φ_r_*_0_) = (1, 0°; 1, 0°), (1.5, 0°; 1.5, 0°), and (3, 0°; 3, 0°) to generate dual-channels FVBs of higher orders, including orders 2, 3, and 6, respectively. Figure 5 shows the corresponding theoretical and simulated results under the illuminating light of horizontal polarization. The above theoretical and simulated results demonstrate that with the spatially multiplexed metasurfaces and by controlling parameters of *φ_l_*_0_, *φ_r_*_0,_ and *q_l_*, *q_r_* of the nanoslits, different dual-channel FVBs may be generated. The polarization states of the FVBs can be determined by both the initial orientation angle of the nanoslit and the linear polarization direction of the illuminating light. Here, we note that the distribution and evolution of phase and polarization states of the VBs as given in the results are related to the interesting topological phenomena such as Möbius polarization state and dark intensity links [47,48].

## 4. Experimental Setup and Measurement Results

**Figure 6:**
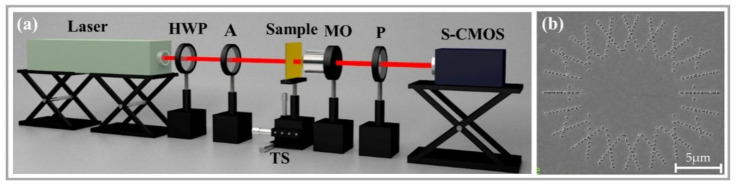
(**a**) Schematic diagram of the experimental setup. (**b**) SEM image of Sample 5.

We experimentally verified the method of generating dual-channel FVBs using the proposed metasurface. Six samples were fabricated on the Au films with 200 nm thickness over silica substrates using focused ion beam etching. Table 1 shows the parameters (*q_l_,* *φ_l_*_0_; *q_r_*, *φ_r_*_0_) of the samples and the small-sized scanning electron microscopy (SEM, FEI Co., Hillsboro, OR, USA) images, with the enlarged views of the nanoslits given in center of the images. Figure 6a illustrates the experimental setup, and Figure 6b shows the normal-sized SEM image of sample 6 as an example. In Figure 6a, a He-Ne laser (Shanghai Hongyang Inc., Shanghai, China) of wavelength *λ* = 632.8 nm was used as the linearly polarized light source. The polarization direction of the illuminating light was adjusted by the half-wave plate (HWP, Daheng Inc., Beijing, China), and the attenuator (A, Daheng Inc., Beijing, China) was used to adjust the power of the incident light. The sample was placed on the 3D transitional stage. The generated dual-channel FVBs were observed at the focal plane behind the sample and were magnified through a microscope objective lens (MO, NA = 0.9/100). The polarization component patterns of the FVBs were obtained through the analyzing polarizer (P, Daheng Inc., Beijing, China) and were captured by S-CMOS camera (Zyla 5.5, 16 bit, 2560 × 2160 pixels, pixel size 6.5 × 6.5 μm, Andor, Belfast, UK).

**Figure 7:**
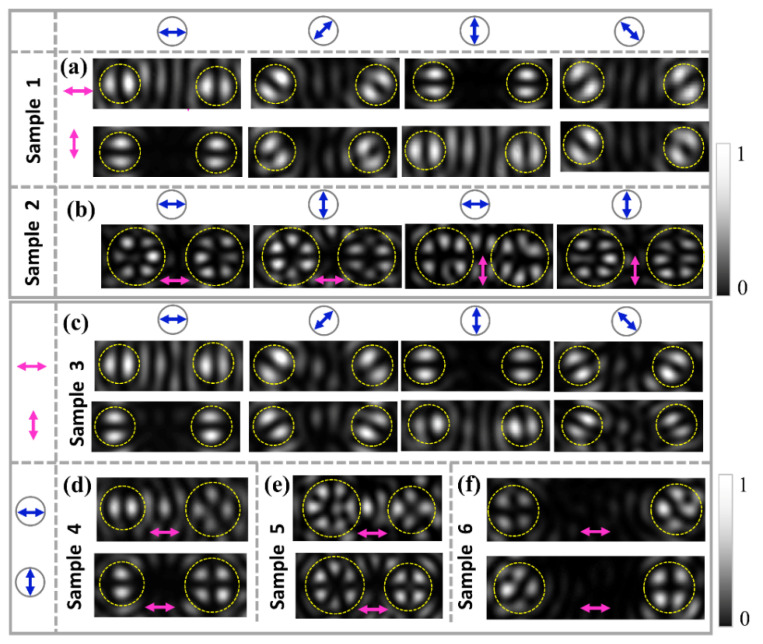
The experimental intensity patterns of different dual-channel FVBs. (**a**) The dual-channel FVBs of orders *l_l_* = *l_r_* = 1 for the Sample 1 under illumination of horizontal and vertical polarization, respectively. (**b**) The dual-channel FVBs of orders *l_l_* = *l_r_* = 3 for Sample 2 under illumination of horizontal polarization. (**c**) The FVBs of order *l_l_* = 1 and *l_r_* = −1 for Sample 3 under illumination of horizontal and vertical polarization, respectively. (**d**–**f**) The dual-channel FVBs of orders *l_l_* = 1 and *l_r_* = 2 for Sample 4, *l_l_* = 3 and *l_r_* = 2 for Sample 5, and *l_l_* = *l_r_* = 2 for Sample 6, respectively, under illumination of horizontal polarization. The magenta double arrows represent the linear polarization of illumination light, and the blue double arrows in circles indicate the transmission axis of the analyzing polarizer P.

The results of the experimental demonstration for generating the dual channel FVBs are shown in Figure 7. By using the horizontally and vertically polarized light to illuminate Sample 1, respectively, the radially and azimuthally polarized dual-channel FVBs of order *l_l_* = *l_r_* = 1 are generated, and the obtained intensity patterns are shown in Figure 7a. The petals of intensity patterns of generated FVBs in the two channels rotate in the same direction with the rotation of the polarizer P. The distribution direction of the petals of the radially polarized FVBs is parallel to direction of the polarizer, while for the azimuthally polarized FVB, the distribution direction of the petals is perpendicular to that of the polarizer P. Figure 7b gives the radially and azimuthally polarized dual-channel FVBs of orders *l_l_* = *l_r_* = 3 generated by Sample 2, respectively, under illuminations of horizontal and vertical polarizations. Figure 7c–e gives the intensity patterns of FVBs with different orders *l_l_* ≠ *l_r_* in the two channels for Samples 3–6, respectively, under the illuminations of horizontally polarized light. In Figure 7c, the intensity patterns of FVBs are for Sample 3 with *l_l_* = 1 and *l_l_* = −1, where the patterns in the upper row are the radial and π-radial FVBs in the left and right channels, respectively, generated under illumination of the horizontal polarization, and the patterns in the lower row are the azimuthal and π-azimuthal FVBs in left and right channels, respectively, generated under illumination of the vertical polarization. Again, the intensity patterns manifest that the petals of radial and π-radial FVBs rotate in the same and the opposite directions, respectively, with the rotation of the analyzing polarizer P, and this is also true for the petals of azimuthal and π-azimuthal FVBs. Figure 7d gives intensity patterns for the radially polarized FVBs of orders *l_l_* = 1 and *l_r_* = 2 in dual-channels for Sample 4, under illumination of horizontal polarization. Figure 7e shows the radial FVBs of higher orders *l_l_* = 3 and *l_r_* = 2 in dual-channels generated by Sample 5. Figure 7f shows the azimuthal and radial FVBs of order *l_l_* = *l_r_* = 2 in the left and right channels, respectively, generated with the Sample 6 under illumination of horizontal polarization. Overall, All the experimental results are consistent with the theoretical and FDTD simulation results, which demonstrates the feasibility of the designed metasurface for generating dual-channel FVBs.

## 5. Conclusions

We propose a spatial multiplexing metasurface composed of nanoslits arranged on the confocal ellipses for generation of independently controlled dual-channel FVBs. In principle, the geometry of the constant sum of distances from a point on ellipse to the two foci is essentially used. The two groups of nanoslits are alternately arranged at the in-phase points and out-of-phase points on the ellipses. By the destructive interference resulted from the propagation phase, the ISC in the output field is eliminated. Meanwhile, by the constructive interference due to the combination of the propagation phase and the geometric phase, the CSC in the output field is focused at two foci. Each set of the in-phase and out-of-phase nanoslit groups is designed for the corresponding focal point in the observation plane with distance *z*; two orthogonal circularly polarized vortices with opposite topological charges are formed at the focal point under LP illumination, their equal-weighted superposition is realized, and generation of the FVB is achieved. With two sets of nanoslits interleaved on all the ellipses, different dual-channel FVBs are generated at the two focal points in the observation plane. Based on the theoretical analyses, the FDTD simulations and the experimental demonstrations, the feasibility of our method for designing the metasurface to generate the dual-channel FVBs is well validated. Obviously, the independent control of the parameters for each channel greatly enhances flexibility for the generation of the FVBs with this method. This work would be of significance to broaden the applications of VBs in the frontier areas such as dynamic polarization optics [22,23,24,25,26,27,28], particle acceleration [6,49], information transmission [40], optical encryption [5], and quantum experiments [8,9,10].

## Figures and Tables

**Table 1:** Parameters of Designed Samples and SEM images.

Sample	1	2	3	4	5	6
Left channel, *q_l_*	0.5	1.5	0.5	0.5	1.5	1
Left channel, *φ_l_*_0_	0°	0°	0°	0°	0°	45°
Right channel, *q_r_*	0.5	1.5	−0.5	1	1	1
Right channel, *φ_r_*_0_	0°	0°	0°	0°	0°	0°
SEM images	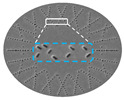	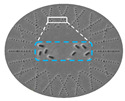	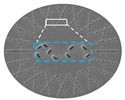	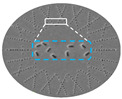	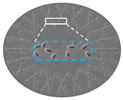	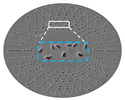

## Data Availability

Data is contained within the article.
